# Photocatalysis of Metallic Nanoparticles: Interband vs Intraband Induced Mechanisms

**DOI:** 10.1021/acs.jpcc.3c04436

**Published:** 2023-08-04

**Authors:** Pin Lyu, Randy Espinoza, Son C. Nguyen

**Affiliations:** † Department of Chemistry and Biochemistry, University of California, Merced, 5200 North Lake Road, Merced, California 95343, United States

## Abstract

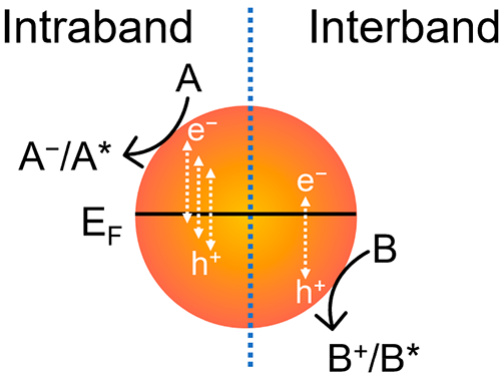

Photocatalysis induced by localized surface plasmon resonance of metallic nanoparticles has been studied for more than a decade, but photocatalysis originating from direct interband excitations is still under-explored. The spectral overlap and the coupling of these two optical regimes also complicate the determination of hot carriers’ energy states and eventually hinder the accurate assignment of their catalytic role in studied reactions. In this Featured Article, after reviewing previous studies, we suggest classifying the photoexcitation via intra- and interband transitions where the physical states of hot carriers are well-defined. Intraband transitions are featured by creating hot electrons above the Fermi level and suitable for reductive catalytic pathways, whereas interband transitions are featured by generating hot d-band holes below the Fermi level and better for oxidative catalytic pathways. Since the contribution of intra- and interband transitions are different in the spectral regions of localized surface plasmon resonance and direct interband excitations, the wavelength dependence of the photocatalytic activities is very helpful in assigning which transitions and carriers contribute to the observed catalysis.

## 1 Introduction

### 1.1 Overview of Metallic Nanoparticles for Catalysis and Photocatalysis

As excellent electron reservoirs, metals are capable of donating or accepting electrons for facilitating chemical transformations. Their macroscopic forms have been used as heterogeneous catalysts for decades, and their nanocrystals have recently gained more attention for exploring new catalytic properties.^1,2^ Metallic nanocrystals were initially used in photocatalysis when they were loaded on TiO_2_ as cocatalysts.^3^ Since then, the heterojunctions of metal and semiconductor nanocrystals have been used extensively for photocatalysis.^4^ They have mostly been prepared by precipitation or impregnation of metal nanocrystals on supporting metal oxide nanocrystals. As a result, the metal nanocrystals have poor uniformities of size and shape and consequently ill-defined catalytic sites. These factors create a challenge on quantifying the relationship between the structure and catalytic properties.^5^ Recently, the development of colloidal synthesis has allowed us to create better quality metallic nanocrystals with high confidence over controlling size, shape, crystal facet, uniformity, and surface functionalization.^6−8^ This advancement now provides unprecedented opportunities to study and tune their catalytic properties with greater accuracy and reliability.

When studying photocatalytic mechanisms of metallic nanocrystals, we have relied on the well-known photophysics of bulk metals and photochemistry at metal surfaces.^9−11^ This is largely because we treat the nanocrystals as bulk materials and consider their electronic states as continuous and metal-like as long as their size is larger than a few nanometers.^12,13^ However, the photophysics of bulk metals cannot always be applied to nanocrystals due to many influencing factors originating from their small dimensions and high curvature. Among these factors, the most distinct properties of metallic nanocrystals that set them apart from their bulk versions are their optical tunability and the corresponding photogenerated hot carriers. The two optical regimes include localized surface plasmon resonance (LSPR) and direct interband transitions (IBs), and they are generally accessed by choosing suitable irradiation wavelengths. As discussed in more detail in Section 2.1, these two regimes offer different electronic transitions. The application of LSPR for photocatalysis has been demonstrated for more than a decade and reviewed thoroughly in literature.^14−19^ Recent attention on utilizing IBs for photocatalysis has brought some promising results, thus diversifying the catalytic mechanisms and their potential application to various reactions.^20−26^

**Scheme 1:**
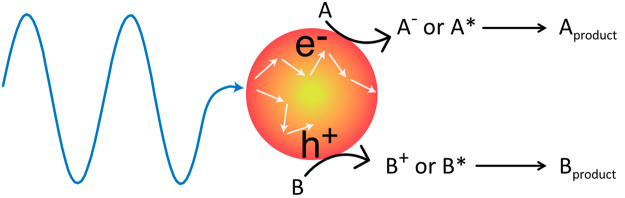
Photocatalysis of Metallic Nanocrystals Discussed in This Featured Article Reactants A and B can undergo charged or excited states to form products. The terms “catalysis” and “photocatalysis” used in this Featured Article are referred to any possibilities of creating new reaction pathways. They are not limited to the traditional definition of catalysis where the reaction pathways are maintained but the activation barriers are lowered.

The purpose of this Featured Article is to compare the photophysics and corresponding catalysis of these two optical regimes, highlight the physical states of the photogenerated hot carriers, and eventually suggest their suitable application for chemical reactions. We introduce the generation and properties of hot carriers under inter- and intraband transitions, then select some representative reactions, discuss the previous photocatalytic mechanisms, propose the new one for interband transitions, and suggest the suitable catalytic pathways (Scheme 1). We hope that this understanding will help in the development of better metallic nanocrystal-based photocatalysts.

### 1.2 Metallic Nanoparticles for Photocatalysis: Pros and Cons

For decades, metallic nanocrystals have demonstrated their potential for photocatalyzing various chemical reactions.^24,27−29^ Regardless of their advantages and disadvantages as compared the well-known molecular^30−35^ and semiconductor photocatalysts^36−39^ (Table 1), metallic nanocrystals have contributed significantly to the catalysis toolbox, and their photocatalytic properties are still relatively new for further exploration. The development of metallic nanocrystal photocatalysts will help to diversify and expedite the applications of photochemistry.

**Table 1:** Compared Photocatalytic Properties of Metallic Nanocrystals to Other Common Photocatalysts

	Metallic nanocrystal	Molecule	Semiconductor nanocrystal
Pros	Tunable light absorption	Better quantum yield	Adjustable band gap
	Tuning hot carrier energy by light	Metal-atom economy	Band alignment for extracting carriers
	Continuous electronic state	Well-defined active site and catalytic mechanism	Long lifetime exciton
	Hot carrier-induced catalysis	Tunable selectivity	Good recyclability
	Near field-induced catalysis	Long lifetime excited state	
	Good recyclability		
Cons	Low quantum yield	Poor recyclability	Utilizing photons is limited by band gap
	Capping ligands hinder catalytic activity	Poor stability	Low quantum yield
	Interior atoms are not efficiently used		

A rough comparison between photocatalysts is presented in Table 1, but a more quantitative comparison can be withdrawn for specific catalysts. For example, metallic nanocrystals have higher recyclability than molecular photocatalysts, but most of their interior atoms do not participate in the photocatalysis, making the nanocrystals still less economical than the molecular catalysts in terms of utilizing materials down to atomic levels. The time window for extracting charge or energy also depends heavily on each kind of photocatalysts. For example, photoredox molecular catalyst Ir(ppy)_3_ has an excited-state time constant of about 2 μs,^32^ while photoexcited semiconductor nanocrystal CsPbBr_3_ can have the charge-extracted lifetime of 40 ns when methyl viologen is used as an electron acceptor.^39^ For noble metal nanocrystals, this window is about tens of femtoseconds when the carriers are still hot (see details in Section 2.2).^11^ The extremely short lifetime of the hot carriers poses a challenge in using them for catalysis.

### 1.3 Charge Transfer in Photochemistry of Bulk Metals: A Starting Point for Studying Photocatalysis of Metallic Nanoparticles

**Figure 1:**
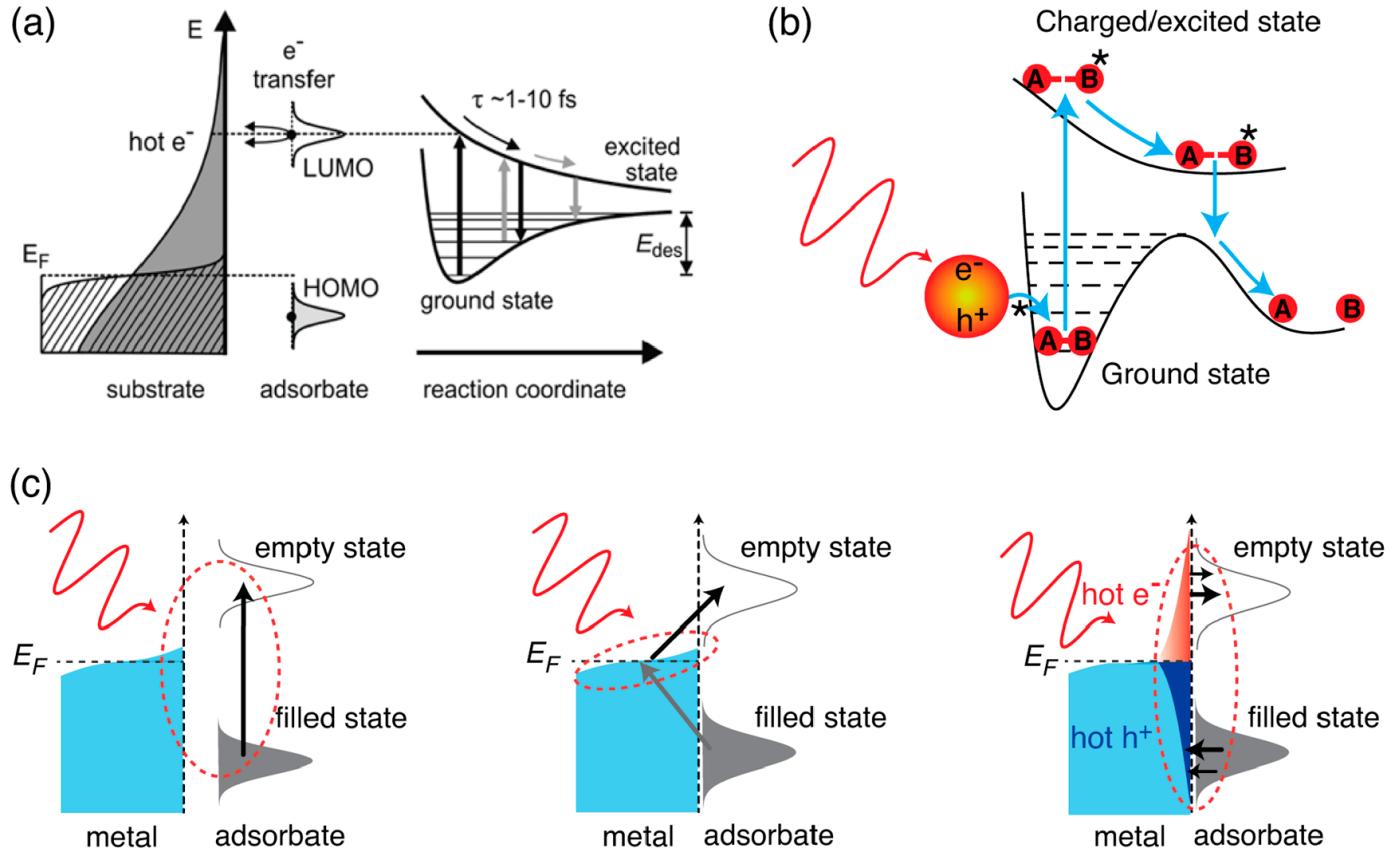
Charge and energy transfer from bulk metals to adsorbates for photocatalysis. (a) The photogenerated hot electrons in bulk metals transfer to the high potential energy surfaces of the adsorbates and facilitate the desorption. Reprinted from ref (10) with permission. Copyright 2006, American Chemical Society. (b) The mechanism in (a) can be applied to nanocrystals, and an activation barrier can be overcome. (c) Some charge and energy transfer mechanisms for exciting adsorbates at the surfaces of bulk metals, such as direct intramolecular excitation (left), direct excitation of hybridized metal–adsorbate states (middle), and indirect hot-carrier injection (right). Modified from ref (44) with permission. Copyright 2020, Wiley-VCH Verlag GmbH & Co. KGaA, Weinheim.

Before discussing on the photocatalytic mechanisms of metallic nanocrystals, it is important to note that the surface photochemistry^9^ and surface femtochemistry,^10^ concerning photoreactions and dynamics of adsorbates on bulk metals, have established charge and energy transfer mechanisms between adsorbates and metals under light irradiation.^40^ For example, photogenerated hot carriers at metal surfaces can transfer charge or energy to the adsorbates (or reactants) and promote them to charged or electronically excited potential energy surfaces (PESs). The adsorbates then can relax to the low-lying PESs and proceed to the next reaction steps, such as desorption or dissociation (see illustration in Figure 1a).^9,10,41^ Apparently, this knowledge for bulk metals has been very helpful to establish the photocatalysis of metallic nanocrystals.^28,42,43^Figure 1b illustrates photocatalysis involving charge or energy transfer that brings a reactant to a charged or excited PES that can overcome an activation barrier.

More detailed mechanisms were proposed for the excitation of reactants at the metal surfaces, such as direct intramolecular excitation, direct excitation of hybridized metal–adsorbate states, and indirect hot-carrier injection (Figure 1c).^44−46^ In brief, direct intramolecular excitation occurs when a surface plasmon resonance directly induces electronic transitions in the adsorbates (Figure 1c, left). The optical field creates a direct coupling of the metal and adsorbate states,^40^ and there is no metal–adsorbate charge transfer. When there is a strong hybridization between metals and adsorbates, charge transfer can occur and promote a direct excitation on the adsorbates (Figure 1c, middle).^47,48^ This particular direct excitation, known as chemical interface damping, happens during the plasmon dephasing time, and the oscillation of the conductive electrons promotes charge transfer between the metals and adsorbates. These two mechanisms (Figure 1c, left and middle) do not necessarily require hot carrier generation in the nanocrystals. Lastly, the indirect hot-carrier injection mechanism needs the formation of hot carriers, and then they are scattered to the surfaces and injected to the adsorbates (Figure 1c, right). These three mechanisms were often proposed in plasmon chemistry of metallic nanocrystals and for the associated electron transfer.^18,19,28,44,49,50^ As we will discuss later, the last mechanism should also work for IBs and for extracting hot holes. Many strategies for extracting hot holes have been recently summarized,^51,52^ encouraging more work on utilizing IBs for photocatalysis.

## 2 Photoexcitation of Metallic Nanoparticles

**Figure 2:**
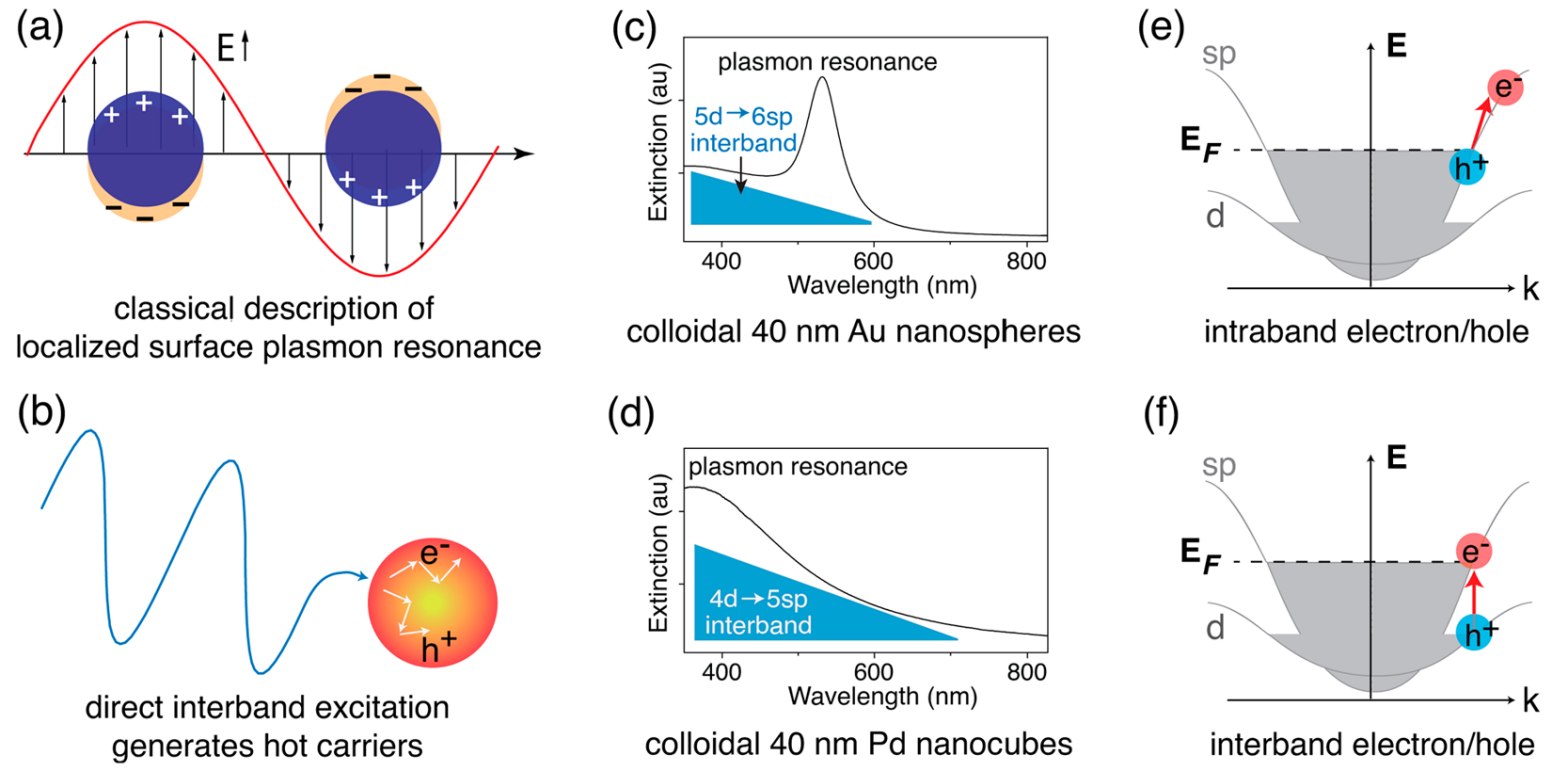
Illustrations of optical properties of metallic nanocrystals and the corresponding hot carrier generation. (a) Conductive electrons of metallic nanoparticles respond collectively to the electric field of resonant photons. (b) Direct interband excitation generates hot carriers across different bands. (c, d) UV–vis spectra of some common metallic nanocrystal photocatalysts show the positions of plasmon resonance and interband transitions. (e, f) Examples of hot carriers generated from intra- vs interband transitions and their corresponding energy levels.

In this section, we systematically compare the LSPR and IBs of metallic nanoparticles in terms of their optical excitations (Figure 2a,b) as well as the energy and dynamics of the hot carriers generated. These properties lay the foundation for the catalytic mechanisms discussed later.

### 2.1 Optical Excitations in Plasmon Resonance and Interband Transition Regimes

The IBs are given electronic transitions from the respective d- to sp-bands, and they are allowed transitions determined by band structures. When these transitions can be accessed directly in the spectral region without any overlap with LSPR, such as around 400 nm in Figure 2c, we often call them direct IBs. The LSPR can be described by a classical picture as a collective oscillation of the nanoparticles’ conductive electrons when they respond to the electric fields of the resonant photons and are restored to their original location (Figure 2a). In the quantum mechanical description, the LSPR promotes electronic transitions from some sp-band states to other sp-band states with different electron momenta; therefore, these are also called intraband transitions (Figure 2e). The decay of LSPR into those forbidden transitions is assisted by phonons, defects, impurities, electron–electron scattering or surface collision.^11^ The LSPR can also include IBs as one of many mechanisms for the plasmon decay and generating hot carriers.^11^ Due to these origins, the LSPR offers strong optical absorptions and tunable spectral shifts that depend on the nanocrystal size, shape, assembly, surrounding environment, and polarization of the incident light. On the other hand, the IBs of each metal always provide an intrinsic absorption within a defined spectral region and are less affected by geometric factors. The LSPR is very strong in gold, silver, copper, or aluminum nanocrystals but becomes weaker in the nanocrystals made of other transition metals.^53^ In palladium or platinum nanocrystal photocatalysts, the IBs contribute largely to their optical absorption (Figure 2d). In general, the LSPR absorption can be tuned from the visible to the infrared regions, but the IBs are mostly available in the shorter wavelength regions ranging from ultraviolet to visible. These two optical regimes have broad extinctions and can be spectrally overlapped. The coupling between them, simply described as the two coupled harmonic oscillators, could be strong,^54,55^ and the LSPR can decay to IBs.^56^ The spectral overlap and coupling between these two regimes cause complications in assigning the photocatalytic mechanisms induced by either of them. This issue will be addressed throughout this Featured Article.

### 2.2 Photogenerated Hot Carriers and Their Properties within Intra- and Interband Transitions

The optical excitations within LSPR or IB regimes are expected to generate hot carriers with different energies, populations, and dynamics. Although their generation can be described by classical or quantum-mechanical pictures, we opt for the latter as it is straightforward to define their energy levels (i.e., their locations in the d- or sp-bands), which are strongly related to the photocatalytic mechanisms. To have a representative comparison between plasmon and interband regimes, gold nanocrystals are demonstrated in the following discussion.

The direct IBs happen when the absorbed photons directly promote electrons from d- to sp-bands (see Figure 2f).^58^ Atwater and co-workers calculated the IB probabilities for various metal thin films and found that the energy distribution of hot carriers strongly depends on their band structures, especially the position of the d-bands.^59,60^ Most energy of the absorbed photons is converted into the potential energy of the hot holes in the d-bands below the Fermi level (*E*_F_), and the hot electrons reside near *E*_F_.^11,61−63^ For gold, the energy gap between the highest occupied 5d and the lowest unoccupied 6sp-bands is about 2.3 eV;^62,64^ thus, only high energy photons of blue or UV light can provide sufficient kinetic energy (on top of the 2.3 eV interband energy gap) to hot carriers, and these high-kinetic carriers can pass the metal–adsorbate energy barrier to catalyze chemical reactions on the nanocrystal surfaces.

The LSPR, on the other hand, is annihilated *via* four mechanisms,^11^ and the IBs can be one of them when the resonant photons have enough energy for those transitions. The other mechanisms are intraband transitions, which happen for the transitions between two states within the same sp-bands. These states must have different momenta; thus, these transitions become allowable by the assistance of phonons, defects, surface collisions or electron–electron scattering.^11,58^ Since the intraband transitions do not require the energy gap like the IBs, the hot electrons can have an energy range from *E*_F_ to *E*_F_ + *ℏ*ω, and the hot holes can reside below *E*_F_ down to *E*_F_ – *ℏ*ω, where *ℏ*ω is the energy of the resonant photon (Figure 2e). These hot electrons can have kinetic energies higher than those generated from IBs. For gold nanospheres, both hot electrons and holes reside in the 6sp-band, and the plasmon resonance peak is around 2.3 eV, which strongly overlaps with the IBs (Figure 2c).

As the LSPR has multiple decay channels including both inter- and intraband transitions, it is quite challenging to define the properties of hot carriers. Besides, the spectral overlap between LSPR and IBs also complicates the determination of these properties. Our approach to overcome this issue in catalytic interpretation is defining these properties within inter- or intraband transitions. As mentioned above and illustrated in Figure 2, when the hot carriers are generated from inter- or intraband transitions, their energy levels are distinguishable. Instead of evaluation of the catalytic activities based on the optical transitions (LSPR vs IBs), we now consider which electronic transitions (inter- vs intraband) can contribute to the catalytic activities. As the probability of IBs increases significantly for high energy photons,^57^ the IBs have stronger contribution to the optical absorption than the LSPR for shorter-wavelength regions (Figure 2c and d). This property allows us to perform wavelength dependence of the photocatalytic activities and correlate the observed activities to the inter- or intraband transitions.

**Figure 3:**
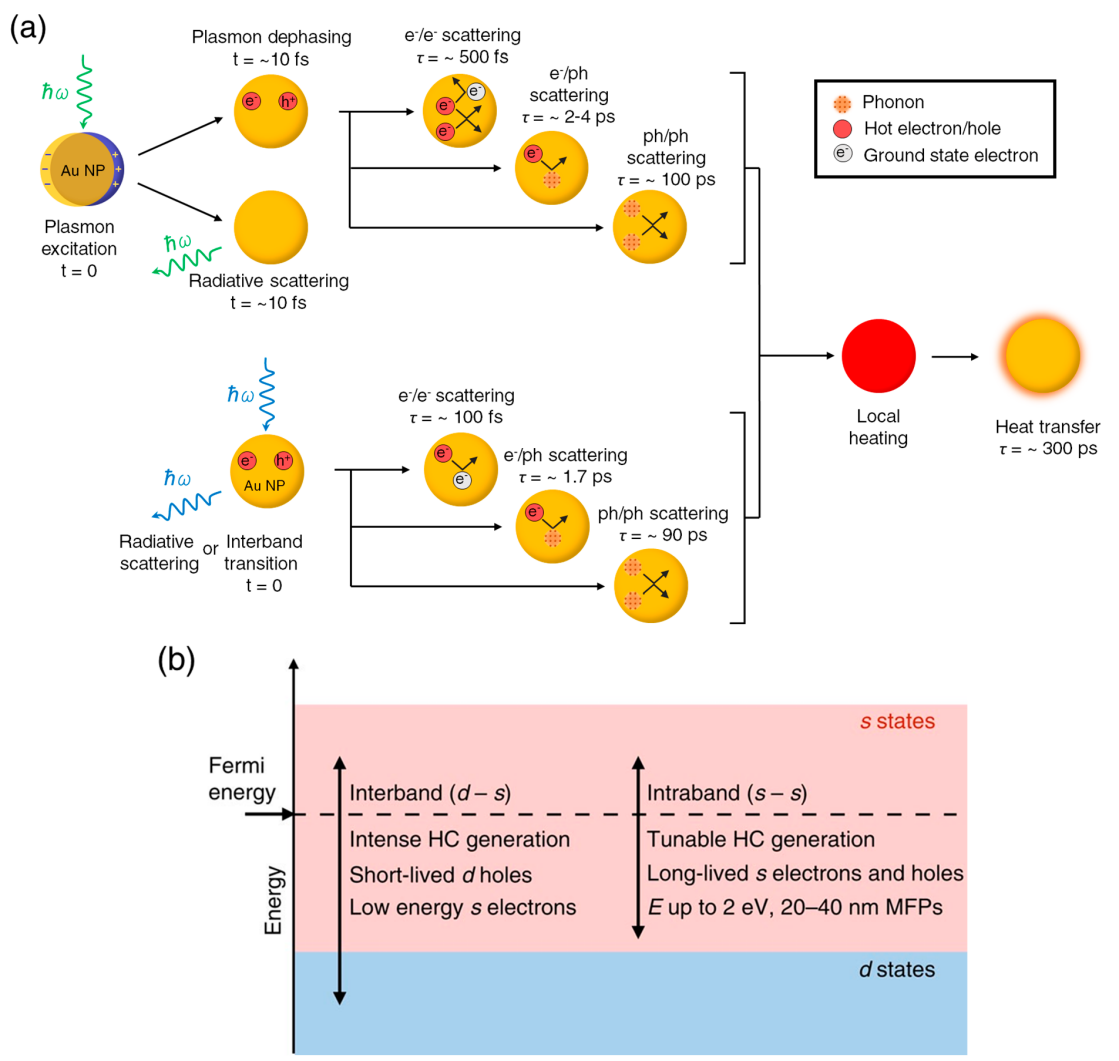
Properties of hot carriers generated from intra- and interband transitions of gold nanocrystals. (a) Time scales (*t*) and time constants (τ) of plasmon resonance (top) and direct interband excitation (bottom) decays and the subsequent relaxation of hot carriers and phonons. (b) Different properties of hot carriers from intra- and interband transitions. HC and MFPs stand for hot carrier and mean free paths. Reprinted from ref (57) with permission. Copyright 2015, The Authors.

Another important factor, besides the aforementioned energy levels, in the utilization of hot carriers is their lifetimes. Figure 3a summarizes the typical time scales for each decay step. Once excited via LSPR, the nanocrystals undergo either nonradiative absorption to generate electron–hole pairs or radiative scattering to re-emit the photons on the time scale of around 10 fs.^65,66^ The hot carrier formation happens within about tens of femtoseconds, which are about the dephasing time of the plasmon^55^ and the time scale of intraband transitions.^65^ The hot carriers, also called primary hot carriers or quasi-ballistic hot carriers, have enough energy to catalyze chemical reactions.^11^ These nonthermal carriers then relax through electron–electron (e-e) and electron–phonon (e-ph) scattering with the time constants of around 500 fs and few picoseconds, respectively.^67−69^ The phonon–phonon (ph-ph) scattering has a time constant of about 100 ps. On the other hand, the interband carriers have a much shorter e-e scattering time due to the fast scattering of electrons into the empty d-band states.^58^ Both e-ph and ph-ph scattering in interband regimes happens with similar time scales as in plasmon resonance. The crystal temperature rises as soon as this scattering starts, and heat transfer to the surrounding environment happens with a time constant of several hundred picoseconds.^70,71^ These time constants were mainly collected from ultrafast spectroscopies in which each nanocrystal absorbed multiple photons within an ultrashort laser pulse (about 100 fs) in order to achieve a significant transient signal for detection. This condition is quite different from continuous-wave irradiation where each nanocrystal roughly absorbs the two consecutive photons within an estimated interval of about a hundred nanoseconds.^72^ This estimation was based on experimental conditions where a high power LED (∼500 mW) was used, and the colloidal solution of the photocatalysts has a high optical absorption (OD = 0.42). Multiple photon absorption in ultrafast spectroscopies results in a higher population of hot carriers, a longer relaxation of the carriers, and a higher local heating as compared to single photon absorption under continuous irradiation. Note that the above time constants are subject to change when the experimental conditions change, such as the excitation or probing wavelengths of laser beams, the size, shape, and capping ligands of the nanocrystals.^55^

Overall, the intraband transitions are featured by hot electrons in the sp-bands with high energy above *E*_F_. The IBs are featured by hot holes in the d-bands, and these holes have a large potential below *E*_F_. These holes have larger effective mass, smaller kinetic energy, shorter mean free paths, and shorter lifetimes than these electrons (Figure 3b).^11,57^ These factors hinder the diffusion of hot holes to nanocrystal surfaces and reduce the efficiency of extracting them for catalysis.

## 3 Photocatalyzed Reactions Driven by Hot Carriers

Despite the debates and concerns about the actual photocatalytic mechanisms and the side effect of local heating on studied reactions,^73−76^ the catalytic role of hot electrons and holes is clear in many reactions. In this section, we highlight some representative cases that lay down the foundation for understanding the photocatalytic mechanisms in Section 4. We categorize the below reactions by the catalytic processes needed for the reactions of interest. For example, we focus on the role of hot electrons (or holes) for the interested reactions, and the hot holes (or electrons) are not our focus even though they may affect the overall catalysis.

### 3.1 Examples of Reactions Catalyzed by Hot Electrons

**Figure 4:**
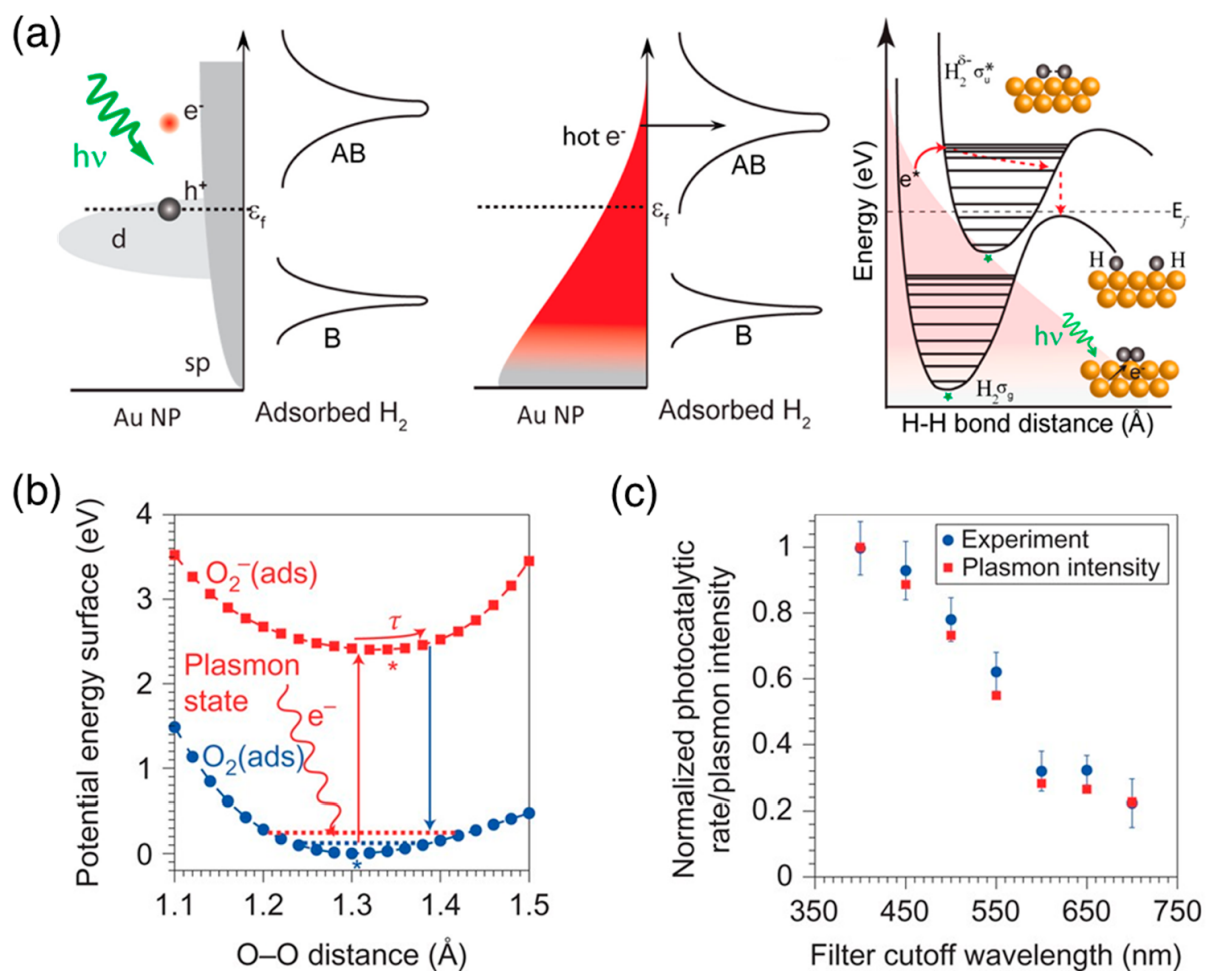
Examples of hot-electron driven photocatalysis. (a) Hot electrons above the Fermi level can transfer to the antibonding state of H_2_ and induce the dissociation on gold nanoparticles. Reprinted from ref (50) with permission. Copyright 2013, American Chemical Society. (b) Excited electrons transferred from silver nanocrystals to O_2_, forming transient O_2_^–^ anion and inducing dissociation. (c) A linear mapping between the normalized photocatalytic rate (blue dots) and total plasmon intensity (red squares) indicates the photocatalytic activity was driven by plasmon excitation and subsequent hot electrons. (b) and (c) reprinted from ref (42) with permission. Copyright 2011, Springer Nature Limited.

As described in Section 2, the hot electrons generated from LSPR have an energy that is above the Fermi level and higher than the ones generated from IBs. Thus, LSPR is more suitable for extracting electrons from nanoparticle photocatalysts to catalyze chemical reactions (Scheme 1). This reductive catalytic pathway has been demonstrated beautifully in previous studies and reviewed recently by Wei,^19^ Moores,^18^ Zhan, Moskovits and Tian,^77^ Xu and Nam,^78^ Jain^79^ and co-workers. Proof-of-concept experiments were demonstrated for activating small molecules, including H_2_, O_2_ and H_2_O dissociation. Nordlander, Halas and co-workers reported the room-temperature dissociation of H_2_ on gold nanoparticles under visible light irradiation. The plasmonic hot electrons were suggested to transfer to the antibonding state of H_2_ and induce the dissociation (Figure 4a).^50^ Linic and co-workers demonstrated the hot electrons in silver nanocrystals could transfer to antibonding orbitals of O_2_, form a transient negative-ion state and facilitate O_2_ dissociation (Figure 4b).^42^ The photocatalytic rates normalized by the plasmon intensity of the silver nanocrystals track well the crystals’ LSPR spectra, supporting hot-electron-driven catalysis (Figure 4c). Note that the IBs of silver starts at around 3.8 eV; thus, the observed photocatalytic activities in the visible region are attributed to the hot electrons formed by intraband transitions.^80^

Meng and co-workers performed DFT simulations for small gold nanoparticles and suggested that the photocatalyzed H_2_O dissociation could be optimized by adjusting the laser intensity and plasmonic hot electrons’ energy levels for efficiently transferring charge to the antibonding orbital of water.^81^ Some related experimental results have been reviewed by Nam,^82^ Cortés^83^ and co-workers. A large portion of organic reactions photocatalyzed by hot electrons were reviewed in previous reports.^18,78,84^ These examples are quite helpful for establishing the photocatalytic mechanisms in Section 4 because the properties of the hot electrons can be correlated to the reaction products. We also see that the indirect hot-electron-injection mechanism (Figure 1c) has been used very often in many photocatalytic interpretations. Note that the hot electron injection can be transient as long as it can induce chemical transformation.^28^ In other words, the net charge transfer to the reaction products is not required, and the photocatalyzed reactions can be redox or nonredox type.

### 3.2 Examples of Reactions Catalyzed by Hot Holes

While the hot-electron mediated reactions have been well demonstrated, the hot-hole mediated ones have recently emerged.^51,52,72^ Hot holes generated from LSPR can catalyze metal etching,^25^ organic transformations,^85−87^ polymerization,^88^ and oxygen evolution reactions.^89^ The hot holes generated from IBs have much lower potentials below the Fermi level and lower energy than those generated from LSPR. In principle, these interband hot holes should be better used to support the oxidative catalytic pathways in which the metal crystals accept electrons during the course of photocatalysis. This approach has been demonstrated for oxidation reactions or other reactions have the oxidation step as the rate-determining step.^24−26,72,90,91^ In this section, we highlight some of those reactions and the catalytic role of “deep” holes from IBs. The term “deep” refers to the very low potential below *E*_F_.

**Figure 5:**
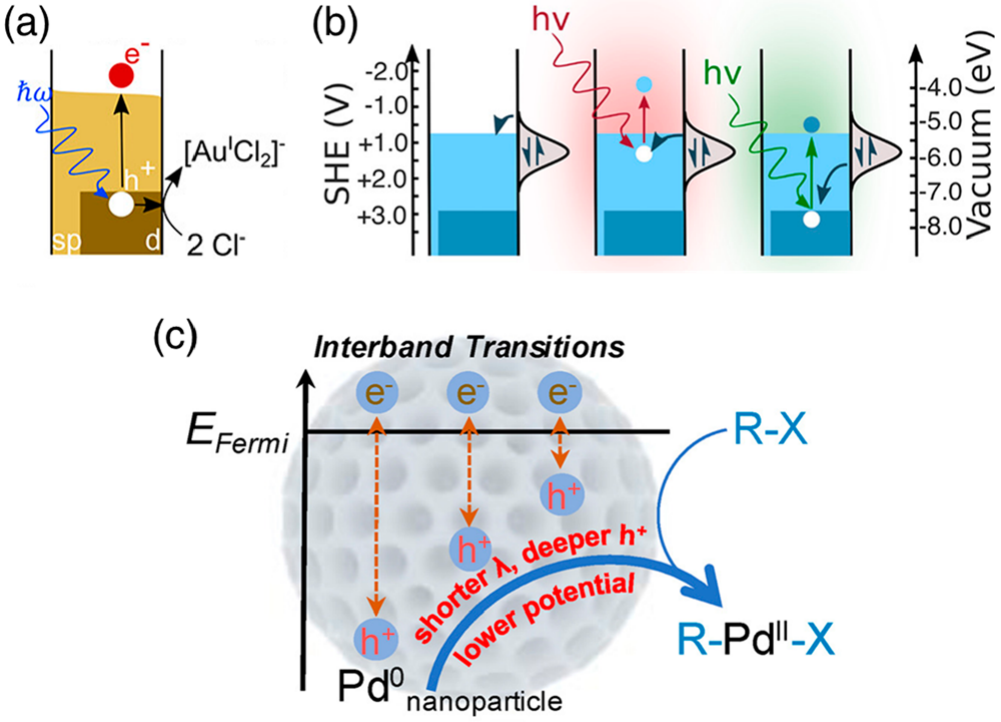
Examples of hot-hole-driven photocatalysis. (a) Photo-oxidative dissolution of individual gold nanorods on indium tin oxide electrodes was enhanced by hot holes. Reprinted from ref (91) with permission. Copyright 2023, American Chemical Society. (b) Illustrations of intra- and interband transitions resulting in the hot holes residing in the sp- and d-bands. The latter significantly catalyzes the oxidative dissolution of the gold nanocrystals described in (a). Reprinted from ref (25) with permission. Copyright 2019, American Chemical Society. (c) Deeper hot holes in the d-bands generated from shorter-wavelength excitations promote better the oxidative addition to form the R–Pd^II^–X intermediate, which results in better catalyzing the Suzuki–Miyaura reaction. Reprinted from ref (72) with permission. Copyright 2022, The Authors.

Starting with the chemistry of hot holes generated from LSPR, Link, Ren, Landes and co-workers demonstrated the photo-oxidative dissolution of individual Au nanorods on indium tin oxide electrodes was enhanced by the plasmonic hot holes (Figure 5a,b).^25,91^ They also compared the dissolution rates under LSPR and IBs and concluded that the d-band holes catalyzed the reaction better.^25^ Note that in this photoelectrochemical cell setup, the electrons on the nanoparticle photocatalysts are channeled through the contact electrodes. Earlier reports by Toste, Alivisatos and Somorjai,^24^ Jain^27^ and co-workers also found that IBs in gold nanoparticles give better photocatalytic activities for reduction of Fe^3+^, indicating the catalytic role of electrons. However, hole scavengers play a critical role in these experiments as they modulate the reduction reaction rates. Our group also found a similar trend of higher catalytic activities of IBs for hydrogenation of styrene by gold nanoparticles.^26^

As mentioned in Section 2.1, the LSPR-IB spectral overlap can be tricky to assign the correct energy levels of the hot holes and their exact contribution to the photocatalytic mechanisms. To reduce this overlap and focus on the photocatalytic properties of the d-band hot holes, our group utilized mesoporous palladium nanocrystals whose LSPR is shifted to the near-infrared region, but their IBs are in the visible.^72^ The deeper holes generated from shorter-wavelength excitation with stronger oxidation power catalyze better the oxidation addition of aryl halide onto the palladium surface (the rate-determining step of the Suzuki–Miyaura reaction, Figure 5c), thus offering a higher product yield.^72^ This work demonstrated the catalytic role of the hot holes, but not the hot electrons as usually suggested,^92^ in the photocatalyzed Suzuki–Miyaura reactions. Note that all of the proposed mechanisms mentioned here are based on the correlation between the hot-carrier properties and the observed products. Ideally, a direct probe of fundamental steps during the course of photocatalysis is still needed to strengthen the proposed mechanism, such as ultrafast spectroscopy to detect charge or energy transfer from the nanocrystals to the reactants.

### 3.3 Examples of Catalyzed Reactions that Need Both Hot Electrons and Hot Holes

**Figure 6:**
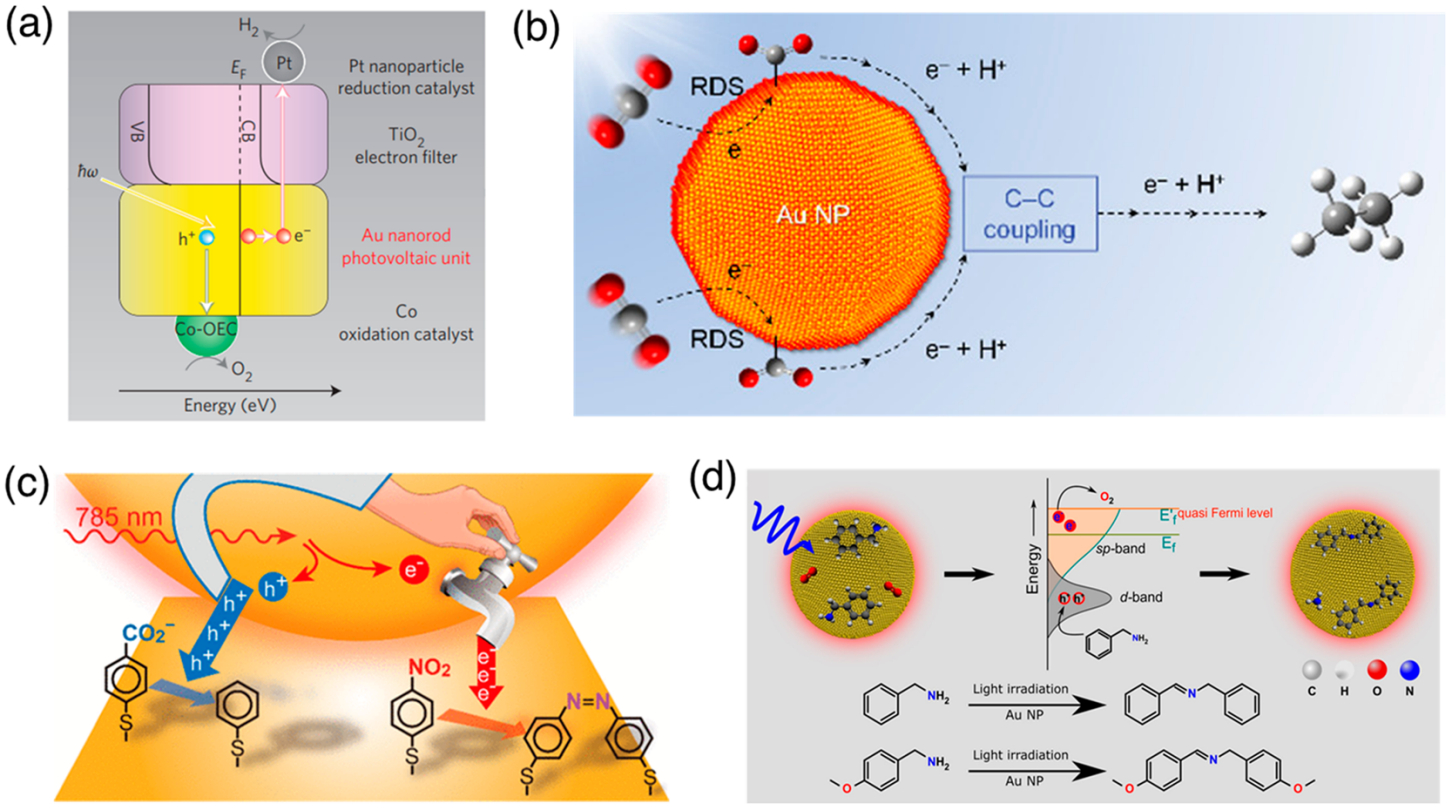
Examples of catalyzed reactions with contributions from both hot carriers. (a) Extraction of energetic hot electrons from gold nanorods to TiO_2_ layers and Pt nanoparticles for hydrogen ion reduction. The holes are filled by electrons from the cobalt-based oxygen evolution catalyst; the overall processes create water splitting. Reprinted from ref (93) with permission. Copyright 2013, Springer Nature Limited. (b) Photocatalyzed CO_2_ reduction to C_1_ and C_2_ hydrocarbons on gold nanocrystals in aqueous solutions. The hot d-band holes meditate the dissociation of the O–H bond in H_2_O (not shown), which then provides electrons and protons for subsequent CO_2_ hydrogenation and C–H formation. Reprinted from ref (94) with permission. Copyright 2018, American Chemical Society. (c) The hot-hole extraction is needed to balance with the hot-electron extraction for reduction of 4-nitrobenzenethiol. Reprinted from ref (85) with permission. Copyright 2020, American Chemical Society. (d) Benzylamine oxidation to *N*-benzylidenebenzylamine mediated by hot electrons and holes. Reprinted from ref (87) with permission. Copyright 2021, Wiley-VCH GmbH.

In many reactions, the extraction of both hot carriers is more suitable, and many groups have been developing different strategies to improve the overall catalytic performance. Moskovits and co-workers proved that the hot electrons from LSPR of gold nanorods are extracted at Au-TiO_2_ interfaces and captured by decorated platinum nanoparticles for hydrogen ion reduction (Figure 6a).^93^ After this extraction, the leftover holes are filled with electrons from the cobalt-based oxygen evolution catalyst. The simultaneous actions of the electrons and holes result in the splitting of water. Jain and co-workers used the hole quenchers to accumulate a net charge on gold nanoparticle photocatalysts, which raises their *E*_F_ for photocatalyzing reduction reactions,^79^ such as CO_2_ reduction to C_1_ and C_2_ hydrocarbons^94^ (Figure 6b). In those aqueous CO_2_ reduction experiments, the hot holes mediating the dissociation of the O–H bond in H_2_O is the rate-limiting step of the entire reaction, and the subsequent CO_2_ hydrogenation and C–H formation are the downstream steps.^95^ The wavelength survey on the photocatalytic activities inferred that the d-band holes generated from IBs played the key role in the rate-limiting step.^95^ Recently, our group quantified that the hole quenchers can scavenge the hot holes efficiently and accumulate a significant amount of negative charge on the nanoparticle photocatalysts, and these photocharged particles can still catalyze reduction reactions as a background phenomenon even when they are not undergoing photoexcitation.^96^

Yoon and co-workers found that both electron- and hole-transfer channels needed to be balanced for the reduction of 4-nitrobenzenethiol at the nanogaps between gold nanocrystals and thin films (Figure 6c). The hot hole promoted the decarboxylation of 4-mercaptobenzoic acid as an electrical switch to balance the charge transfer and turn on the reduction of 4-nitrobenzenethiol.^85^ Obviously, this strategy has been used widely in photocatalysis driven by carriers. Hole or electron quenchers are needed to extract charge from the nanocrystals and reduce the electron–hole recombination within the crystals. A similar mechanism was also reported by Chandra, Rao and co-workers for the oxidative coupling of benzylamine into imine (Figure 6d).^87^

### 3.4 Descriptors of Photocatalysis Efficiency

Quantifying the efficiency of the photocatalysts is an important criterion for mechanistic studies and practical applications, as it allows us to compare their activities under different experimental conditions and estimate the effective cost of using photons for the catalyzed reactions. As recommended by the International Union of Pure and Applied Chemistry,^97^ the best description for the efficiency (i.e., activity) of a photocatalyst is its quantum yield (QY) in a reaction, which is defined as the ratio of the number of product molecules to the number of absorbed photons. As the photocatalysts are activated after absorbing photons, scattered photons are not counted in the QY calculation. Hence, the apparent quantum efficiency, defined as the ratio of product molecules to incident photons, is sometimes used, but light scattering from the catalysts must be taken into account because it lowers the efficiency of utilizing the incident photons. The apparent reaction rates, product yields, or reactant conversions can also be used to compare the relative photocatalytic activities under different experimental conditions. Note that turn over frequency is more suitable for quantifying catalysis efficiency under nonirradiated conditions.^98,99^

**Figure 7:**
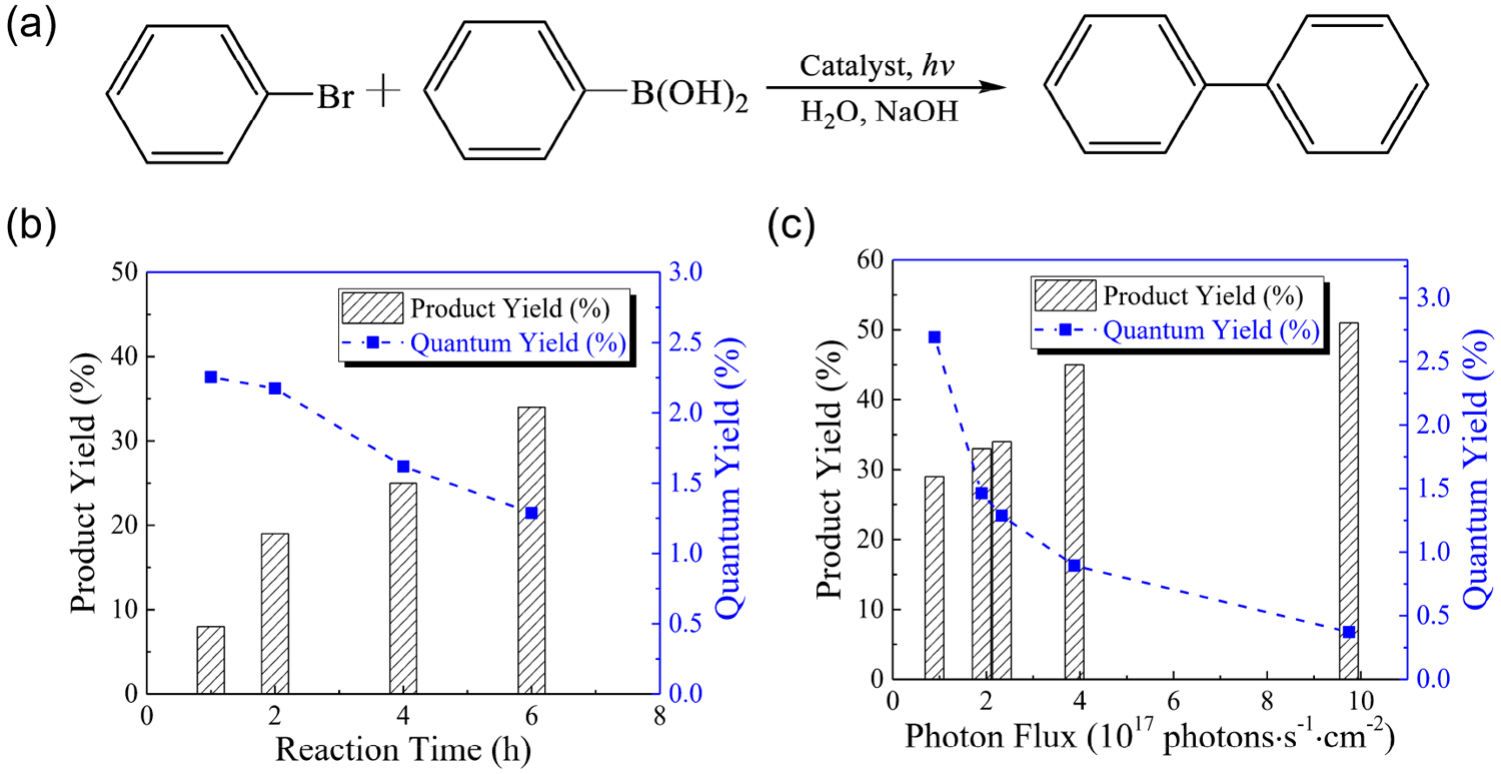
Change of quantum yield (QY) depending on reaction time and photon flux. (a) C–C coupling reaction for evaluating the effect of reaction time and photon flux on the QY of Pd nanoparticle photocatalysts. (b, c) Product yield increases but QY drops when increasing reaction time or absorbed photon flux. Reprinted from ref (72) with permission. Copyright 2022, The Authors.

When the above QY definition is used in the context of monitoring a photocatalyzed reaction, the QY is calculated as the number of product molecules produced within a specific time divided by the number of photons absorbed during that time. This means that the numerator is the measured reaction rate and the denominator is the optical power absorbed by the photocatalysts. Since the reaction rate decreases as the reaction proceeds but the photon absorption rate is unchanged, the QY drops over a longer monitoring time. Similarly, the QY also drops when a higher photon flux is used and absorbed by the catalysts because each activated photocatalyst effectively has fewer reactants to catalyze. This phenomenon was demonstrated for a reaction with palladium nanoparticles (Figure 7).^72^ Hence, when comparing the QY under different experimental conditions, it is important to remember that the QY depends on reaction time and photon flux. This dependence should be accounted for when comparing QY under interband or plasmon excitations or even across different photocatalysts.

## 4 Photocatalysis Mechanism of Hot Carriers in Metallic Nanoparticles

With the typical examples of photocatalyzed reactions mentioned in Section 3 and the photophysics of metallic nanoparticles in Section 2, we now summarize and propose the photocatalytic mechanisms mediated by hot carriers. Note that many other important mechanisms are not covered in this Featured Article, such as near-field enhancement,^100−102^ photocharging,^79,96,103^ and photothermal effect.^71,72,104−110^

### 4.1 Mechanisms Induced by Intraband Transitions

**Figure 8:**
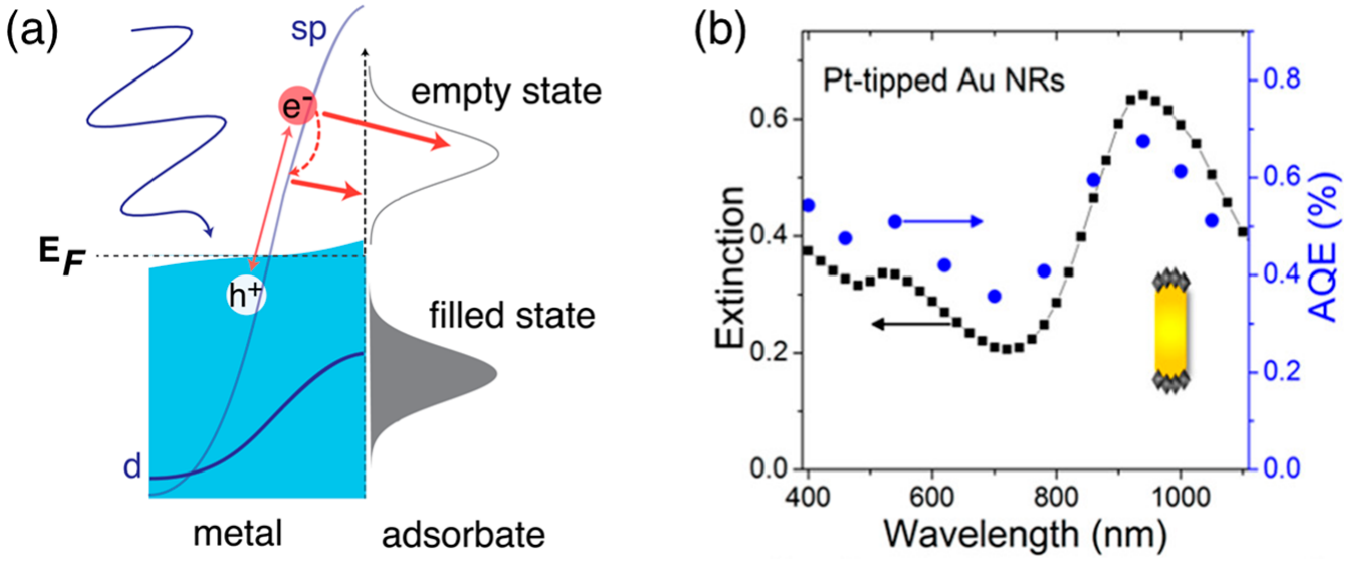
Photocatalysis mechanisms induced by hot electrons from intraband transitions. (a) Electron transfer for the reductive catalytic pathway. (b) Wavelength dependence of apparent quantum efficiency (AQE) for the hydrogen evolution from a water–methanol mixture. The catalysts are plasmonic platinum-tipped Au nanorods (inset). The mapping of AQE to nanoparticle extinction and the high AQE at long wavelengths indicate the contribution of intraband transitions to the catalysis. Reprinted from ref (111) with permission. Copyright 2014, American Chemical Society.

As mentioned earlier, the hot electrons from intraband transitions have higher energy than the Fermi level and can transfer to the empty states of the reactants (Figure 8a). This mechanism is often called indirect electron transfer since the hot carriers must first be generated before being transferred to the reactants. This interpretation is indeed borrowed from the indirect hot-carrier injection mechanism for bulk metals (Figure 1c, right). As shown in many examples in Section 3.1 and Figure 4, the transferred electrons can activate chemical bonds by filling the antibonding orbitals. The stronger electronic coupling between the reactants and nanocrystals, the easier this transition occurs. Since the hot electrons quickly lose their energy through e-e and e-ph scattering, this relaxation significantly reduces their probability of reaching the nanocrystal surfaces and transferring to the reactants. Thus, the diffusion of hot carriers to and through nanocrystal surfaces to reach the reactants plays a critical role in catalytic efficiency.^26^ Lastly, the wavelength dependence of the photocatalytic performance is very useful to evaluate the contribution of intraband transitions and the corresponding hot electrons to the catalytic mechanism. As illustrated in Figure 8b, the mapping of the apparent quantum efficiency (AQE) to the photocatalysts’ extinction and the high AQE at long wavelength (below the interband threshold at 2.3 eV or 539 nm) indicate the catalytic role of intraband transitions and hot electrons.^111^

Some other photocatalytic mechanisms do not require the formation of hot carriers but work under LSPR. It is important to highlight them here. When there is a strong electronic coupling between the nanocrystals and the reactants (e.g., strong orbital hybridization between them), the LPSR decay can cause a direct electron transfer, which is in a fashion similar to a direct excitation in bulk metals (Figure 1c, middle). As the nanocrystals have large surface-area-to-volume ratios, chemical interface damping (CID) causes a significant decay of plasmon resonance, and the charge transfer through CID happens without necessarily forming hot carriers. Consequently, tuning the plasmon resonance can excite specific electronic states of the reactants and activate specific catalytic pathways.^46^ Furthermore, the direct photoexcitation of the hybridized adsorbate–metal states can also happen and give high product selectivities.^112,113^ In this case, the nanocrystals play the role of the substrates, which mediate the direct photoexcitation of the adsorbates.

The mechanisms described above highlight the benefit of utilizing energetic plasmon electrons for catalysis (Figure 8a). The reductive catalytic pathways, in which the reactants or intermediates receive electrons, are preferred. However, the hot holes can also participate in some oxidative pathways.^114,115^ Another mechanism that is not discussed in this Featured Article is the plasmon-induced resonance energy transfer, mostly observed in metal–semiconductor heterojunctions.^116^ As opposed to the well-known Förster resonance energy transfer, the plasmonic energy transfers toward the high energy direction to induce charge separation in metal–semiconductor heterojunctions.^117^ This mechanism may also support energy transfer from metallic nanocrystals to reactants and trigger catalytic processes.

### 4.2 Mechanisms Induced by Interband Transitions

**Figure 9:**
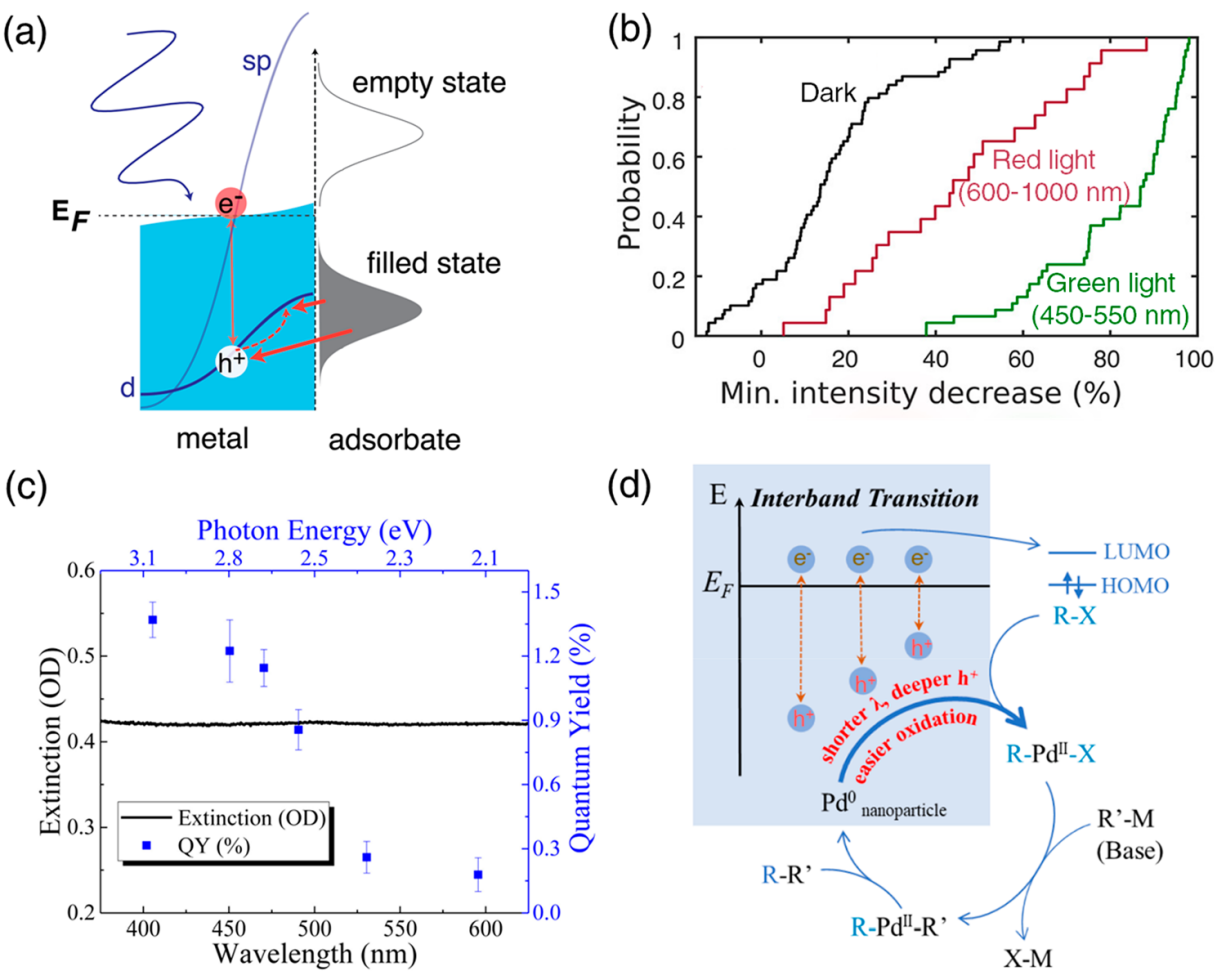
Photocatalysis mechanisms induced by hot holes from interband transitions (IBs). (a) Hole-filling mechanism for oxidative catalytic pathways. (b) The reduction of scattering intensity of gold nanoparticle dissolution under red and green light irradiation. The latter promotes more IBs, resulting in more d-band hot holes for better catalysis. See more information in Figure 5a,b. Modified from ref (25) with permission. Copyright 2019, American Chemical Society. (c, d) Deeper holes from short-wavelength excitation can catalyze the Suzuki–Miyaura reaction by accelerating oxidative addition of aryl halide R–X onto Pd^0^ at the crystal surface. The quantum yields are much higher for IBs. Reprinted from ref (72) with permission. Copyright 2022, The Authors.

Photocatalysis induced by IBs has been less commonly exploited. As described in Section 2.2, the signature of IBs is the hot holes residing in the d-bands below the Fermi level. These holes with a strong oxidizing power can be filled by electrons from the reactants. Thus, IBs should be applied to oxidative catalytic pathways. The hot holes can be quenched by electrons from the filled states of the adsorbates. This mechanism is favored by the strong metal–adsorbate electronic coupling and the high energy levels of the adsorbate filled states. Those hot holes can also relax to higher energy states in the d-bands due to the fast electron filling of the metals from e-e scattering,^58^ and then later filled by electrons from the adsorbates (Figure 9a). We predict that the hole transfer is strongly supported when the filled states have more d-orbital characteristics like the d-band holes.

Link, Landes and co-workers illustrated that a large number of hot holes in the d-band can accelerate the oxidative dissolution of gold nanocrystals better than the holes generated from intraband transitions.^25^ The wavelength dependence of the photocatalytic activities is again quite helpful for the catalytic assignment of IBs and hot holes (Figure 9b). The green light must have more IBs transition than the red light, and the dissolution of gold is matched with the role of deep holes in the d-band.

In our previous work, porous Pd nanoparticles were used to shift the LSPR to the near-infrared region, and the photocatalytic activities were studied only under interband transitions in the visible region. It was demonstrated that the deeper holes generated from shorter-wavelength excitation accelerate better the rate-determining step of the Suzuki–Miyaura reaction (Figure 9c,d).^72^ So far, our proposed mechanism follows the indirect charge transfer mechanism (Figure 9a), while the direct charge transfer is a subject of future studies. As mentioned in Section 2.2, these hot holes have larger effective mass and shorter lifetimes than the hot electrons; thus, their extraction efficiency is low. Our porous nanostructure increases the probability of hot carrier diffusion to the catalyst’s surfaces, thus offering a higher photocatalytic efficiency as compared to the nonporous Pd nanoparticles in the same reaction condition.

## 5 Summary and Future Perspectives

Throughout this Featured Article, we show that most of the proposed mechanisms for metal nanoparticle photocatalysts are built from surface photochemistry and surface femtochemistry of bulk metals. Noticeably, intraband transitions can offer hot electrons above the Fermi level and are generally suitable for reactions with reductive catalytic pathways. Interband transitions can create hot holes in the d-bands below the Fermi levels and are better used for reactions that are catalyzed through oxidative pathways. As the intra- and interband transitions often have different contributions to the optical spectra of the LSPR and IBs, wavelength dependence of catalytic efficiencies (or activities) can be correlated with the behavior of hot carriers, which helps to distinguish these two contributions. Besides, the fundamental steps of the well-known reactions are also important to assign the role of hot electrons or hot holes in observed catalysis.

Combining LSPR and IBs for harvesting both energetic hot electrons and deep holes in a precisely integrated system is promising and already shows some prominent results from Frei and Alivisatos,^118^ Chen and Liu,^119^ Sá and Atwater^120^ and co-workers. Multicomponent and multifunction systems with precise control and extraction of both hot carriers are important for improving photocatalytic performance.

One remaining important challenge in understanding the onset and elemental steps of photocatalysis is the direct observation of charge or energy transfer from photoexcited metallic nanocrystals to reactants. The reactants exchange charge or energy with the nanocrystals during the course of photocatalysis, but experimental data for these processes are not yet fully available.

As IBs in noble metal nanoparticles have recently gained more attention for photocatalysis due to the unique properties of the d-band hot holes, it is desirable to explore them in non-noble metal nanoparticles for future applications as these transitions are available in many transition metals. Expanding the mechanisms featured here to other affordable metals will help us move from precious to earth-abundant metals for developing cost-effective and sustainable photocatalysts.
